# Therapeutic Efficacy of Indole‐3‐Carbinol Against SARS‐CoV‐2‐Induced Acute Respiratory Distress Syndrome: A Dual Antiviral and Anti‐Inflammatory Approach in a Golden Syrian Hamster Model

**DOI:** 10.1111/jcmm.71213

**Published:** 2026-07-13

**Authors:** Centofanti Federica, Rizzacasa Barbara, Latini Andrea, Biancolella Michela, Pocci Marco, Servadei Francesca, Scimeca Manuel, Mauriello Alessandro, Bernadett Palyi, Daniel Deri, Berenike Novak, Zoltan Kis, Claudia Filippone, Pier Paolo Pandolfi, Novelli Giuseppe

**Affiliations:** ^1^ Department of Biomedicine and Prevention Tor Vergata University of Rome Rome Italy; ^2^ Department of Molecular Biotechnology and Health Sciences University of Turin Turin Italy; ^3^ UniCamillus Saint Camillus International University of Health Sciences Rome Italy; ^4^ Department of Biology Tor Vergata University of Rome Rome Italy; ^5^ Anatomy and Pathological Histology Unit Veneto Institute of Oncology IOV‐IRCCS Padua Italy; ^6^ Department of Experimental Medicine, and Tor Vergata University of Rome Rome Italy; ^7^ National Center for Public Health and Pharmacy National Biosafety Laboratory Budapest Hungary; ^8^ European Research Infrastructure on Highly Pathogenic Agents, ERINHA AISBL Brussels Belgium; ^9^ ERINHA Central Coordinating Unit Paris cedex 13 France

**Keywords:** hamster, HECT E3 ligase inhibition, indole‐3‐carbinol, preclinical study, SARS‐CoV‐2

## Abstract

SARS‐CoV‐2 has caused a global pandemic, resulting in over two million deaths and creating an urgent need for effective treatments. Severe COVID‐19 is frequently complicated by respiratory failure and acute respiratory distress syndrome (ARDS), the primary drivers of mortality. Indole‐3‐Carbinol (I3C), a natural compound derived from *Brassicaceae* that acts as an inhibitor of HECT family E3 ubiquitin ligases, exhibits potent anti‐SARS‐CoV‐2 activity and inhibits viral egress. However, its in vivo therapeutic efficacy against SARS‐CoV‐2‐induced lung injury remains unproven. We evaluated the therapeutic efficacy of I3C in reducing the severity of SARS‐CoV‐2 infection and associated lung lesions using the Syrian golden hamster ( *Mesocricetus auratus* ) model, which recapitulates the acute lung injury observed in human COVID‐19. Treatment with a non‐toxic dose of I3C (2 mg) significantly ameliorated disease across all parameters, reducing weight loss, improving clinical symptom scores and *reducing histopathological lung damage observed post‐mortem*. A significant reduction in pulmonary TNF‐α levels accompanied this. These findings indicate that I3C mitigates COVID‐19‐related morbidity at clinically relevant, non‐toxic doses. Given its dual antiviral and anti‐inflammatory mechanisms, I3C represents a compelling therapeutic candidate for further clinical investigation.

## 1 Introduction

SARS‐CoV‐2 has caused a global pandemic, resulting in over two million deaths and creating an urgent need for effective treatments [1].

COVID‐19 presents a wide range of clinical symptoms, ranging from asymptomatic or mild conditions to very severe clinical signs [2]. COVID‐19 can be defined first and foremost as a viral‐induced inflammatory disease of the upper airways and lungs, which in some cases can lead to severe respiratory symptoms. It is well known that SARS‐CoV‐2 binds the angiotensin‐converting enzyme 2 (ACE2) receptor to infect cells, leading to viral proliferation [3]. Therefore, the host's induction of inflammatory, innate and adaptive immune responses is sustained to eliminate the virus, but this in turn might cause tissue damage [4]. The most common complications of severe COVID‐19 are respiratory failure and acute respiratory distress syndrome (ARDS), characterized by diffuse alveolar damage, interstitial pneumonitis and lymphocytic infiltrates [5], which represent the main mortality factors for the disease [2].

Therefore, it is crucial to identify molecules that can reduce the infectivity and severity of lesions in individuals with clinical symptoms following SARS‐CoV‐2 infection. To this end, a rapid preclinical evaluation of safe drugs with high antiviral potential in animal models is crucial for verifying their therapeutic efficacy and potential toxicity. Recently, the natural compound Indole‐3‐Carbinol (I3C) has been shown to exhibit potent anti‐SARS‐CoV‐2 activity and to inhibit viral egress [6, 7].

I3C acts as an inhibitor of HECT family members of E3 ligases involved in SARS‐CoV‐2 pathology by physically interacting with and ubiquitylating the SARS‐CoV‐2 spike protein [7]. Importantly, we previously demonstrated that I3C is effective against the SARS‐CoV‐2 Omicron variant, with no toxicity observed, thereby characterizing it as a safe and potentially antiviral compound [8]. These data suggest the potential use of I3C as an antiviral drug in clinical trials for the treatment of COVID‐19 patients.

The golden Syrian hamster ( *Mesocricetus auratus* ) is one of the most widely used animal models, as it is permissive to the replication of several zoonotic viruses, including SARS‐CoV‐1 and 2 and Maporal virus, as well as other viruses with high pathogenic potential [9, 10]. The hamster has proven to be a valuable model for investigating the pathogenesis of these important diseases and for testing new treatments, such as passive immunization or antiviral compounds [11, 12]. Moreover, the hamster is naturally susceptible to SARS‐CoV‐2 infection, requiring no prior adaptation of the virus strains, and can develop pathological features similar to those of human COVID‐19, thus representing an excellent translational model for studying possible therapeutic interventions in the treatment of SARS‐CoV‐2 infections [9].

On this basis, we investigated the therapeutic potential of I3C against SARS‐CoV‐2 infection using a hamster model. Our findings revealed that a 2 mg dose of I3C delayed the onset and shortened the duration of clinical symptoms. Histopathological analysis revealed a reduction in alveolar oedema at Day 4 post‐infection in the 2 mg group, although overall inflammatory scores did not differ significantly. This apparent anti‐inflammatory effect was further supported by substantially lower *TNF‐α* (Tumour Necrosis Factor‐α) mRNA levels in the lungs of the treated animals. Furthermore, serum TNF‐α levels in the 2 mg group remained stable at Day 14, unlike the elevated levels typically seen in progressive infections, suggesting that I3C also mitigates systemic inflammation. Collectively, these results indicate that a 2 mg dose of I3C exhibits both antiviral and anti‐inflammatory properties, positioning it as a promising therapeutic candidate for countering SARS‐CoV‐2‐induced lung pathology.

## 2 Methods

### 2.1 Viruses

The SARS‐CoV‐2 virus delta variant (EPI_ISL_17024327) originated from the National Biosafety Laboratory's microorganism strain collections. The virus was propagated in Vero cells (Nuvonis, Austria) maintained in Virus Production Serum‐Free Medium (VP‐SFM, Gibco, LifeTechnologies, Germany) supplemented with 2X GlutaMAX‐I (Gibco, LifeTechnologies). The 70% confluent cells were infected with the SARS‐CoV‐2 delta variant at a multiplicity of infection of 0.001 and incubated for 5 days at 37°C with 5% CO_2_. The virus‐containing cell culture supernatant was centrifuged at 4500 g for 10 min. A working virus stock was generated from the second passage.

The 50% tissue culture infectious dose (TCID_50_) per mL of the virus stock was determined by an endpoint dilution assay under the same conditions as for propagation. Read‐out based on the presence of cytopathic effect was performed by crystal violet staining after inactivating and fixing the cell culture monolayer in 10 V/V% formaldehyde‐PBS solution.

### 2.2 Ethics Statement

Animal experiments were performed in accordance with the guidelines of the European Communities Council Directive (86/609/EEC). They were reviewed and approved by the Hungarian National Authority (Scientific Ethics Council for Animal Experiments (PE/EA/00107‐6/2024)).

### 2.3 Chemical Treatment

Indole‐3‐Carbinol (I3C) was obtained from Sigma‐Aldrich (Product Number: 17256, CAS‐No.: 700‐06‐1). For in vivo assay, the corresponding dose of I3C suspension was prepared using 10% DMSO, and the solvent was a 0.9% sodium chloride solution.

### 2.4 Animals

The 5–6‐week‐old hamsters were purchased from Janvier Labs (France). All procedures were conducted in the BSL‐3 laboratory at the National Biosafety Laboratory of the National Centre for Public Health and Pharmacy (Budapest, Hungary). The animals were kept in ISORAT900 IV cages (Techniplast, Italy) and were monitored daily. The bedding for the animals was made of corn‐cob litter, and all animal cages included environmental enrichment and non‐pharmaceutical pain alleviation (soft bedding). The animals' feeding and drinking were ad libitum, the feeding was autoclavable full‐value feed (SAFE D30, Safe Complete Care Competence), and the water was autoclaved tap water. The high containment facility centrally regulates temperature and humidity and provides periods of natural light.

### 2.5 Experimental Design for Treatment and Infection of Syrian Golden Hamsters

Twenty‐four male Syrian golden hamsters were treated with I3C (Sigma‐Aldrich) or control buffer (negative control) using the intraperitoneal (IP) route. The animals were treated from 1 day before SARS‐CoV‐2 infection (Day −1) through 4 days post‐infection, as outlined in the experimental plan shown in Table 1. I3C was freshly prepared each day just before administration. The hamsters were infected on Day 0 via the intranasal route with 10^4^ TCID_50_ of the virulent SARS‐CoV‐2 Delta variant strain (EPI_ISL_17024327).

**TABLE 1:** Experimental design for treatment and infection of Syrian golden hamsters.

Arm	Group	Number of hamsters	Treatment dose	Infection with SARS‐CoV‐2 (day)	Euthanasia (day)
Arm 1	A	4	2 mg I3C/hamster	0	4
	B	4	2 mg I3C/hamster	0	14
Arm 2	A	4	4 mg I3C/hamster	0	4
	B	4	4 mg I3C/hamster	0	14
Arm 3	A	4	Vehicle (DMSO)	0	4
	B	4	Vehicle (DMSO)	0	14

Abbreviations: DMSO, dimethyl sulfoxide; I3C, indole‐3‐carbinol.

### 2.6 Clinical Observations

Clinical symptoms, weight and temperature of the animals have been monitored daily from Day −1 to the date of euthanasia using appropriate equipment.

Clinical signs of disease were assessed using a numerical scoring system (points indicated in brackets), based on the literature [11, 12] (Table 2): healthy (0), lethargy (1), behavioural change (1), sunken eyes (2), ruffled (2), wasp‐waisted (3), dehydrated (3), arched (3), coughing (3), laboured breathing occasional catch or skip in breathing rate (5), and laboured breathing abdominal effort with breathing difficulties (7). Animals were weighed at the same time each day until euthanasia.

**TABLE 2:** Scoring system for hamster clinical observations.

Clinical sign	Points
Healthy	0
Lethargy	1
Behavioural change	1
Sunken eyes	2
Ruffled	2
Wasp‐waisted	3
Dehydrated	3
Arched	3
Coughing	3
Laboured breathing occasional catch or skip in breathing rate	5
Laboured breathing abdominal effort with breathing difficulties	7

### 2.7 Harvesting of the Samples

Sampling has been harvested for Group A at Day 4 (euthanasia) and for Group B at Day 14 (euthanasia). The euthanasia was carried out by CO_2_ inhalation after isoflurane inhalation treatment.

### 2.8 Sacrifice, Blood and Organ Collection

For hamsters of groups A, lung and blood samples have been collected at Day 4 before euthanasia; for hamsters belonging to groups B, collection of lung and blood samples has been carried out on Day 14 or when the animals have lost over 20% of their body weight or when severe signs of disease, such as difficulty breathing, have been observed. Lungs have been stored in neutral‐buffered formalin for histopathological assessment.

### 2.9 Histological Evaluation

For histological analysis, lungs from each hamster were fixed in 4% formalin, dehydrated, paraffin‐embedded, sectioned and stained with standard Haematoxylin and Eosin (H&E). All stained sections were digitized (Panoramic Midi II Rx, Epredia) and examined by a pathologist in the 4 mg I3C group, the 2 mg I3C group and vehicle‐treated controls at Days 4 and 14.

A cumulative severity score was assigned to acute inflammatory lesions, along with an estimate of airspace reduction (alveolar consolidation). The scored parameters (cumulative score ranging from 1 to 3) included alveolar oedema, congestion, suppurative bronchitis, bronchopneumonia, perivascular lymphocytic cuffing and alveolar consolidation. The percentage of lung parenchyma involved by inflammatory infiltrates was quantified using AI‐based software (SlideViewer Quant Center; 3D HISTECH).

### 2.10 Total RNA Extraction and Gene Expression

Lungs were homogenized in TRIzol Reagent for RNA extraction according to the manufacturer's instructions. RNA concentration was evaluated using the NanoDrop DS‐11 Spectrophotometer (DeNovix) and RNA quality was assessed on a 1% agarose gel. Next, 1 μg of total RNA was reverse transcribed using the High‐Capacity cDNA Reverse Transcription Kit (Applied Biosystems, Waltham, MA). We analysed the expression of inflammatory‐related genes (*IFNβ*, *CXCL10*, *IL‐6*, and *TNF‐α*); *ACTB* gene was used for data normalization. Real‐time PCRs (RT‐qPCRs) have been performed using ABI7500 Fast Real‐time PCR System (Life Technologies) with Sybr Green Assay (Power Sybr Green PCR Master Mix, Life Technologies) and specific primer pairs (Table S1).

### 2.11 ELISA Test for Hamster TNF‐α Quantification

Serum samples were collected from hamsters from Groups A (at Day 4) and Groups B (at Day 14). The concentration of TNF‐α in the serum samples was determined by Hamster TNF‐α ELISA Kit (EHA0004, FineTest Biotech Inc., Boulder, CO, US) according to the manufacturer's instructions. The microplate was read at O.D. at 450 nm, and then the concentration of TNF‐α in the sample was determined by plotting a standard curve, subtracting the OD450 blank.

### 2.12 Statistical Analysis

Molecular experiments were performed in technical triplicate, and data were analysed using GraphPad Prism 10. A two‐way ANOVA and a one‐way ANOVA test were used to test differences between groups. Significance was set at a minimum of *p* ≤ 0.05.

## 3 Results

### 3.1 Pre‐Treatment Protocol With 2 mg of I3C Significantly Delayed Symptom Onset

**FIGURE 1:**
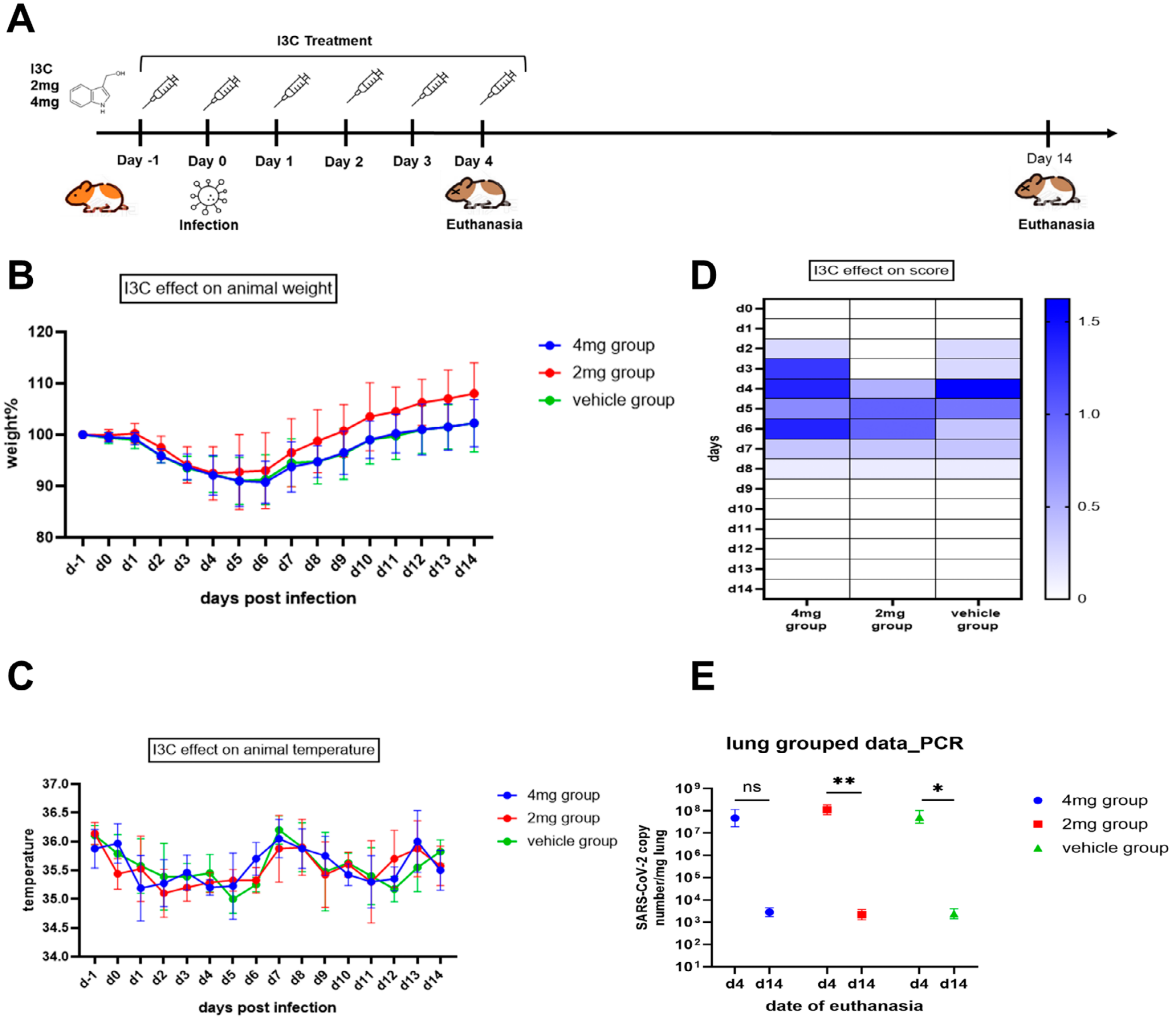
In vivo antiviral activity of I3C against SARS‐CoV‐2. (A) Schematic experimental design. Male Syrian golden hamsters (*n* = 24) were treated intraperitoneally with I3C (2 mg or 4 mg) or vehicle (DMSO) and infected with SARS‐CoV‐2 Delta; monitoring was performed from Day −1 to scheduled euthanasia (Day 4 or 14). (B) Body weight (% of baseline) and (C) temperature according to treatment groups. (D) Average daily scores according to treatment groups. (E) Average SARS‐CoV‐2 copy number/mg lung according to treatment groups (**p < 0.01; *p = 0.05; two‐tailed *t*‐test).

Twenty‐four male Syrian golden hamsters were treated with two different doses of I3C (Sigma) or with a control buffer (negative control, DMSO) via the IP route and infected with the SARS‐CoV‐2 Delta variant (Figure 1A). As none of the animals reached the critical endpoint, only programmed euthanasia was carried out.

The body weight and temperature of each hamster were monitored from the day before (Day −1) treatment initiation until the day of euthanasia. The average body weight and temperature of each group of animals are expressed as a percentage of the initial body weight at Day −1 and shown in Table 3.

**TABLE 3:** Average body weight % by treatment groups.

	Average body weight (as a % of average weight at Day −1)															
	Day −1	Day 0	Day 1	Day 2	Day 3	Day 4	Day 5	Day 6	Day 7	Day 8	Day 9	Day 10	Day 11	Day 12	Day 13	Day 14
4 mg	100%	99%	99%	96%	94%	92%	91%	91%	94%	95%	96%	99%	100%	101%	102%	102%
2 mg	100%	100%	100%	97%	94%	92%	93%	93%	97%	98%	101%	103%	104%	106%	107%	108%
Vehicle	100%	100%	99%	96%	93%	92%	91%	91%	94%	95%	96%	99%	100%	101%	102%	102%

Overall, hamsters treated with 2 mg of I3C showed a trend towards lower average weight loss compared to those treated with 4 mg of I3C or the vehicle group; however, this difference did not reach statistical significance (Figure 1B). The weight of the 2 mg group is higher than that of the vehicle on Days 8 and 9. The greater weight loss in the 4 mg group may be linked to increased compound toxicity. Core body temperature did not differ significantly among the three groups (Figure 1B,C).

Each animal was also clinically assessed daily until euthanasia, using well‐established parameters. Qualitative assessments of clinical signs of disease (excluding weight loss) were scored using an arbitrary, weighted scale, with more clinically significant signs assigned higher values (see Section 2) [11, 12]. Daily clinical scores were then summarized and are presented in Figure 1D as average scores for the treatment groups.

The 2 mg I3C treatment was associated with attenuated disease severity, as evidenced by significantly lower clinical scores during the active infection phase, a delayed symptom onset and a reduced symptomatic period compared with the control groups (Figure 1D). Conversely, the 4 mg group exhibited more severe clinical signs, likely reflecting dose‐limiting toxicity.

### 3.2 I3C Pre‐Treatment Leads to Significant Differences in SARS‐CoV‐2 Copy Numbers in Lungs

At the time of programmed euthanasia, the lungs of each hamster were removed and the SARS‐CoV‐2 copy number and infective titers in the supernatant were assessed. The lung data were grouped according to the day of euthanasia and treatment group (Figure 1E). Significant differences in SARS‐CoV‐2 copy numbers per mg were observed between Day 4 and Day 14 in the 2 mg and vehicle groups (2 mg‐Day 4 vs. 2 mg‐Day 14: **p < 0.01; vehicle‐Day 4 vs. vehicle‐Day 14: *p = 0.05; two‐tailed *t*‐test). Although a similar decrease in SARS‐CoV‐2 copy number was observed in the 4 mg treatment group between Day 4 and Day 14, statistical significance could not be confirmed due to a high standard deviation in the 4 mg group at Day 4.

### 3.3 Pre‐Treatment Protocol With 2 mg of I3C Reduces Early Alveolar Oedema

**FIGURE 2:**
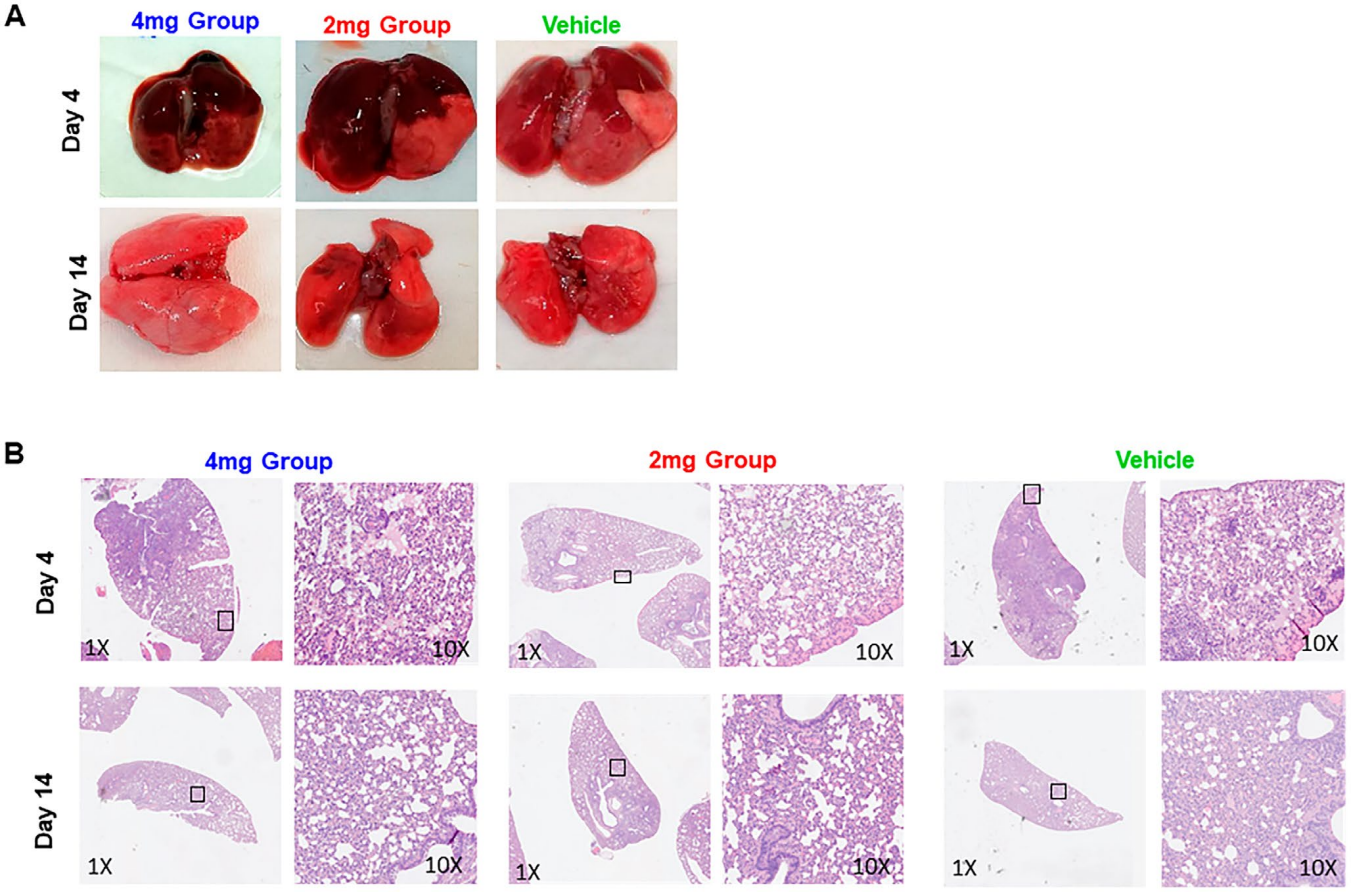
Lung histopathological change after I3C treatment. (A) lung at dissection. (B) Representative images of haematoxylin and eosin (H&E) staining of the lung lobe and alveolar inflammation.

To evaluate lung injury and the development of ARDS in treated and untreated hamsters, histological analysis of lung tissue collected on Days 4 and 14 post‐infection was performed (Figure 2A).

At Day 4 post‐infection, all groups exhibited widespread bronchopneumonia, characterized by suppurative bronchitis and severe mononuclear inflammation in the peribronchial and interstitial areas. Features of acute lung injury, including alveolar oedema, congestion and perivascular lymphocytic cuffing, were also present to varying degrees. The samples consistently showed vascular endothelial atypia and reactive changes, with occasional necrosis of the bronchial epithelium. Although large areas of uninvolved parenchyma remained, early alveolar consolidation was observed in samples from both the treated and vehicle groups. By Day 14 post‐infection, the acute inflammatory component had largely resolved in all groups. However, complete recovery of the lung parenchyma was not observed. Instead, all groups showed alveolar consolidation, resulting in a notable reduction in air spaces. This was characterized by the exuberant presence of mononuclear cells within the air spaces and thickened alveolar septa, consistent with reactive pulmonary cells (pneumocytes) or macrophages. Overall, no significant differences in the histological appearance or the extent of the inflammatory process were observed among the different treatment groups at either time point (Figure 2B).

**FIGURE 3:**
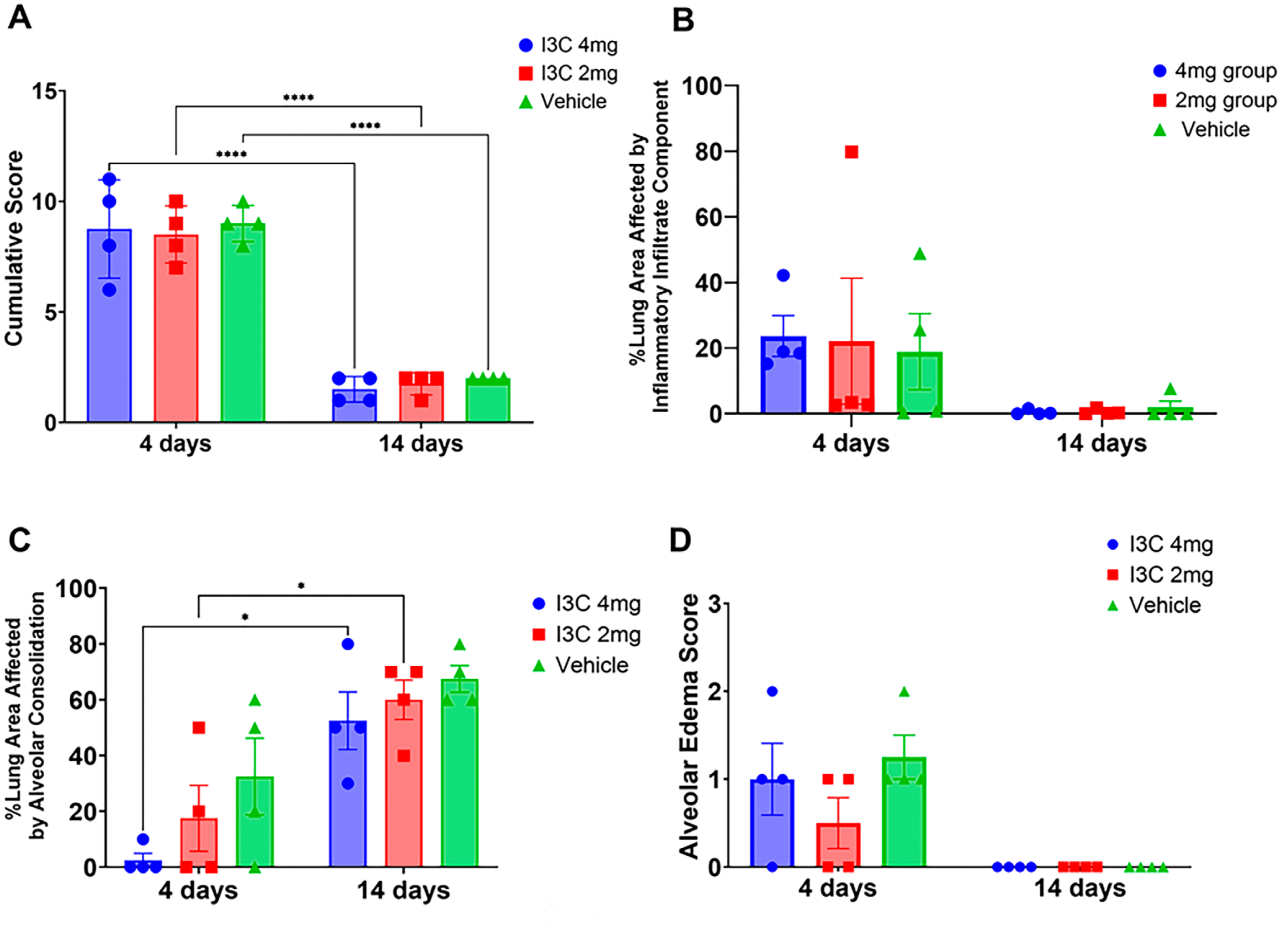
(A) The cumulative histopathology scores. *n* = 4 animals per group. (B) The percentage of area affected by inflammatory infiltrate in the lung sections. 4 mg treated group (*n* = 4), 2 mg treated group (*n* = 4) and SARS‐CoV‐2‐infected hamsters treated with vehicle (*n* = 4), at 4 and 14 days were shown. Horizontal bars represent the mean value of each group ± SEM. Statistically significant differences relative to the vehicle control were determined using a Two‐way ANOVA. (C) The percentage of area affected by alveolar consolidation in the lung sections. 4 mg treated group (*n* = 4), 2 mg treated group (*n* = 4) and SARS‐CoV‐2‐infected hamsters treated with vehicle (*n* = 4), at 4 and 14 days were shown. Horizontal bars represent the mean value of each group ± SEM. Statistically significant differences relative to the vehicle control were determined using a Two‐way ANOVA. (D) Alveolar oedema score. 4 mg treated group (*n* = 4), 2 mg treated group (*n* = 4) and SARS‐CoV‐2‐infected hamsters treated with vehicle (*n* = 4), at 4 and 14 days were shown. Horizontal bars represent the mean value of each group ± SEM. Statistically significant differences against the vehicle control were determined using a Two‐way ANOVA test.

To assess the acute inflammatory lesion, a cumulative score based on the severity of histopathological lesions was assigned to the lung tissue. Although we observed a significant reduction in the cumulative acute inflammatory lesion score from Day 4 to Day 14 across all groups (****p < 0.0001), no significant differences were observed among the three groups (4 mg, 2 mg and vehicle) (Figure 3A).

We also measured the percentage of lung parenchyma area affected by the inflammatory infiltrate (Figure 3B). At 4 days post‐infection, all three groups showed a significant acute inflammatory response, with no significant difference between the 4 mg, 2 mg and vehicle groups. At 14 days post‐infection, we observed that the acute inflammatory component had substantially resolved in all three groups, with a reduction of the area of acute inflammation from Day 4 to Day 14. This indicates that the acute phase of inflammation had passed by Day 14, regardless of treatment. This data also demonstrates that the acute inflammatory component resolves naturally over time in this hamster model of SARS‐CoV‐2 infection.

We also assessed the percentage of lung area affected by alveolar consolidation (reduction in air spaces) in this process (Figure 3C). As expected, at 4 days post‐infection, all groups showed a relatively low percentage of lung area affected by consolidation. The I3C 4 mg group had the lowest average score, followed by the I3C 2 mg and vehicle groups, but the differences were not statistically significant. This indicates that at this early stage, the disease had not yet progressed to widespread lung damage. At 14 days post‐infection, all groups showed a significant increase in the percentage of lung area affected by consolidation compared to Day 4. The vehicle group had the highest average consolidation, followed closely by the I3C 2 mg group. However, the I3C 4 mg group showed a reduction in lung consolidation compared to both the vehicle and the I3C 2 mg groups, but the differences were not statistically significant (Figure 3C). This suggests that while a 2 mg dose of I3C was ineffective at preventing late‐stage lung consolidation, the 4 mg dose showed a protective effect.

Finally, we compared the effects of the two I3C doses (4 and 2 mg) with a vehicle group, assigning a score for oedema severity at 4‐ and 14‐day post‐infection (Figures 2B and 3D).

At 4 days post‐infection, while the reduction in oedema scores did not reach statistical significance, a notable downward trend was observed exclusively in the I3C 2 mg group. Specifically, the 2 mg group showed a higher frequency of lower scores than the vehicle and 4 mg groups, which consistently clustered in the higher‐score range. The reduction in oedema scores at the 2 mg dose suggests that this dosage could modulate the cytokine storm‐driven vascular permeability that characterizes the acute phase.

As expected, at 14 days post‐infection, the alveolar oedema had almost completely resolved in all three groups. This indicates that oedema is primarily a feature of the acute phase of the infection in this hamster model. In particular, the resolution of oedema across all groups by Day 14 confirms that the hamster model accurately captures the transition from acute injury to recovery [13], further highlighting that the critical window for I3C intervention is the early inflammatory phase.

### 3.4 I3C Pre‐Treatment Protocol Leads to a Significant Suppression of Pulmonary TNF‐α Expression

**FIGURE 4:**
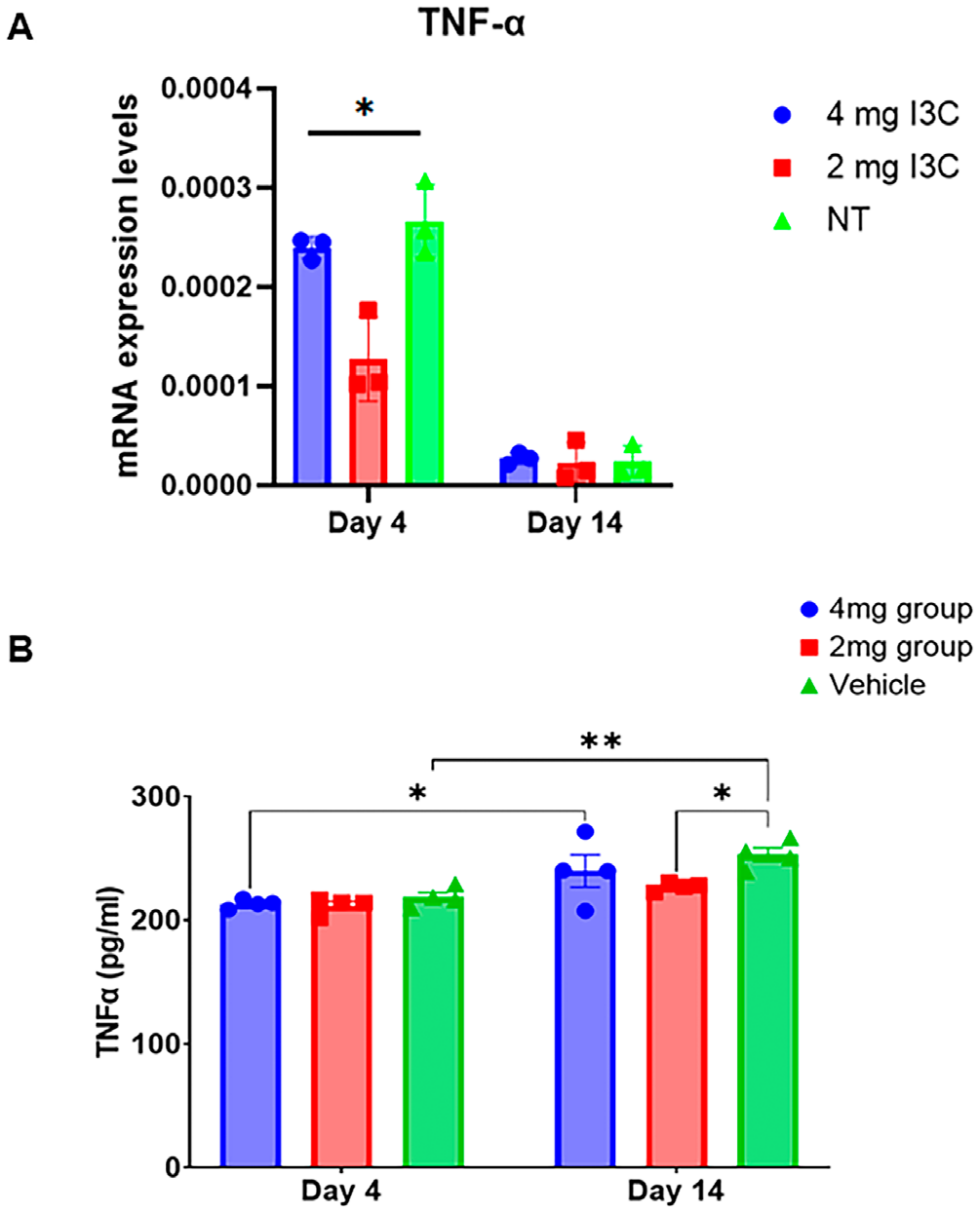
(A) *TNF‐α* mRNA expression study and (B) TNF‐α protein level in the serum of infected hamsters at both Day 4 and Day 14. *p ≤ 0.05, one‐way ANOVA test.

To study the effects of the I3C pre‐treatment protocol on the innate immune response and support its efficacy against SARS‐CoV‐2, we assessed, by RT‐qPCR, the expression of 4 inflammation‐related genes (*IFNβ*, *CXCL10*, *IL‐6 and TNF‐α*) in the lungs of infected hamsters at both Day 4 and Day 14, for each of the three groups analysed. We confirmed that SARS‐CoV‐2 induced the expression of *type I IFN (IFNβ)* and pro‐inflammatory chemokines and cytokines (*CXCL10*, *IL‐6 and TNF‐α*) at Day 4. Interestingly, *TNF‐α* mRNA levels in the group of hamsters treated with 2 mg of I3C were significantly lower compared to the other groups. This data may reflect the lower clinical score observed in the hamsters in this group, as Day 4 appears to be the day of the highest clinical scores during the active phase of the infection. By Day 14, levels of all inflammatory genes analysed had returned to baseline levels, regardless of treatment (Figure 4A).

We also investigated TNF‐α at the protein level in the serum of infected hamsters on Days 4 and 14 post‐infection for each of the three groups analysed. Unlike gene expression data, the ELISA assay performed on hamster sera showed that at Day 4 post‐infection after treatment, TNF‐α levels remained unchanged in both treated and untreated hamsters. Interestingly, at Day 14 post‐infection, we observe a statistically significant increase in serum TNF‐α levels in both the group of hamsters treated with 4 mg of I3C and those only infected with Sars‐CoV‐2 (*p = 0.0136 and **p = 0.0033, respectively). In contrast, serum TNF‐α levels remain unchanged in the group of hamsters treated with 2 mg of I3C. Furthermore, comparing serum TNF‐α levels at Day 14, we observe that levels in hamsters treated with 2 mg I3C are significantly reduced compared to those in hamsters infected with SARS‐CoV‐2 (Figure 4B; *p = 0.02). We hypothesized that 4 mg I3C might elevate TNF‐α levels due to oxidative stress induced by higher I3C doses [14, 15]. This data suggests that, unlike lung tissue, in which *TNF‐α* mRNA levels decreased faster after 14 days, TNF‐α serum levels in the bloodstream remain sustained/high after 14 days (Figure 4B).

## 4 Discussion

The epidemiological trend of SARS‐CoV‐2 nowadays does not allow us to hypothesize a rapid disappearance of the disease, and despite available vaccines, the need for effective therapeutics against SARS‐CoV‐2 remains, particularly to mitigate severe lung pathology. Indole‐3‐carbinol (I3C), a natural compound derived from *Brassicaceae*, exhibits numerous biological properties [16]. We previously demonstrated that I3C significantly reduces SARS‐CoV‐2 viral entry and downregulates the expression of genes involved in innate immune and inflammatory responses in a human lung organoid model [8]. Furthermore, we established that I3C inhibits SARS‐CoV‐2 viral egress in VeroE6 cells by blocking HECT E3 ubiquitin ligases implicated in COVID‐19 pathology, is effective against variants like Omicron and has a favourable safety profile, positioning it as a promising antiviral candidate [7, 8]. The crucial role of E3 ubiquitin ligases is further supported by evidence that SARS‐CoV‐2 hijacks the host ubiquitination machinery to promote viral spread [17]. This strategy is not unique to SARS‐CoV‐2; inhibition of specific ubiquitin‐dependent pathways has been shown to attenuate the manipulation of the host immune response during infections with other viruses, including Influenza, HIV and Dengue [18]. An additional proposed mechanism of action for I3C involves the modulation of the aryl hydrocarbon receptor (AhR), a key regulator of immune and inflammatory responses, suggesting another pathway through which it could ameliorate SARS‐CoV‐2 infection [19]. To evaluate the therapeutic efficacy of I3C in vivo, we used the golden Syrian hamster ( *Mesocricetus auratus* ) model, which closely recapitulates key physiological and pathophysiological features of human SARS‐CoV‐2 infection, including the development of acute lung injury.

The results of this in vivo preclinical study support the therapeutic potential of I3C for the treatment of SARS‐CoV‐2 infection. An integrated analysis of clinical, molecular and histological data suggests that administering a 2 mg dose of I3C can reduce disease severity, attenuate the inflammatory response and promote recovery in infected animals. In contrast, the higher 4 mg dose appears to induce counterproductive toxic effects.

Clinically, the 2 mg I3C group exhibited a non‐significant trend towards reduced weight loss compared to both the 4 mg and vehicle control groups. This suggests that the lower dose may be beneficial, with minimal adverse events. The pronounced weight loss observed in the 4 mg group, however, is likely attributable to compound toxicity at the higher dose. This finding aligns with previous studies indicating that while I3C can modulate immune responses, it may induce undesirable effects—such as metabolic alterations and oxidative stress—at elevated concentrations [14, 20].

Although overall histopathological lung scores and the extent of the inflammatory infiltrate did not differ significantly among groups at 4‐ or 14‐day post‐infection, a specific reduction in the alveolar oedema score was observed in the 2 mg I3C group compared to the vehicle control. This suggests that a 2 mg dose of I3C may specifically mitigate the development of alveolar oedema during the early, acute phase of SARS‐CoV‐2 infection. This is a critical finding, as alveolar oedema is a key pathological driver of acute lung injury and ARDS [15]. The clinical significance of reducing alveolar oedema is essential. In the lungs, oedema impairs gas exchange by flooding the alveoli, directly compromising respiratory function [21]. The attenuation of oedema observed with I3C treatment is therefore highly relevant, as it likely contributes to improved oxygenation and clinical outcomes. The pathophysiology of this oedema in acute lung injury is primarily driven by increased vascular permeability [22]. Pro‐inflammatory cytokines and chemokines, such as TNF‐α, damage the endothelial cells lining the pulmonary capillaries, leading to fluid leakage into the interstitium and alveoli [23]. TNF‐α is a critical driver of acute lung inflammation, promoting immune cell infiltration, vascular permeability and apoptosis of epithelial and endothelial cells. Its elevated levels are a hallmark of severe COVID‐19 and ARDS [23]. Our evaluation of inflammatory markers confirmed that treatment with 2 mg I3C significantly reduced TNF‐α mRNA levels in lung tissue at Day 4 post‐infection. Since TNF‐α is a key mediator of the cytokine storm, this reduction suggests a primary mechanism by which I3C protects against acute inflammatory lung damage, a finding consistent with studies linking cytokine modulation to improved clinical outcomes [24, 25]. This anti‐inflammatory property of I3C is further supported by the work of Liu et al., who demonstrated that I3C protects against myocardial injury by decreasing the expression of *TNF‐α*, *IL‐1β* and *IL‐6*, highlighting its broad anti‐apoptotic, antioxidant and anti‐inflammatory effects [26]. The significantly lower *TNF‐α* mRNA levels in the 2 mg group at Day 4 likely explain the improved clinical scores observed during the peak of active infection.

Interestingly, the anti‐inflammatory effect of I3C appears to extend beyond the local lung environment. While *TNF‐α* mRNA in lung tissue decreased more rapidly over time, analysis of the systemic circulation revealed a statistically significant reduction in serum TNF‐α levels in the 2 mg I3C group at Day 14 post‐infection. This prolonged reduction in a key systemic cytokine suggests that I3C may have a sustained effect in controlling the systemic inflammatory response, which is often associated with protracted COVID‐19 morbidity. The dynamic changes in TNF‐α levels observed between the lungs and serum likely reflect the spatiotemporal progression of the immune response. Initially, elevated TNF‐α in the lungs represents a localized effort to control the infection by recruiting immune cells and initiating an inflammatory response. As the infection resolves by Day 14, local production decreases to prevent collateral tissue damage. However, systemic inflammation can persist, leading to sustained serum TNF‐α levels, potentially due to ongoing immune activation or the release of cytokines from other affected tissues [27, 28, 29]. The clinical relevance of sustained systemic TNF‐α is underscored by studies in tuberculosis, where higher serum levels correlate with greater radiological lung damage, weight loss, symptom severity and worse clinical outcomes [30]. Collectively, our data demonstrate that a 2 mg dose of I3C exhibits both anti‐inflammatory and antiviral properties, thereby ameliorating SARS‐CoV‐2‐induced lung pathology. The favourable safety profile and efficacy of this easily achievable dose support its strong potential for further preclinical development as a therapeutic strategy for COVID‐19.

In conclusion, the observation that I3C modulates both the acute phase (oedema trends) and the inflammatory resolution (TNF‐α levels) suggests that its mechanism of action is likely host‐directed rather than virus‐specific. By targeting conserved cellular pathways involved in viral egress and assembly, such as the NEDD4 and WWP1 ubiquitin ligases or the AhR pathway, I3C may act as a broad‐spectrum antiviral agent. Our findings demonstrate that I3C possesses dual antiviral and anti‐inflammatory properties, effectively countering SARS‐CoV‐2‐induced lung injury. This hypothesis supports the idea that I3C could be repurposed for other respiratory viruses that share similar etiopathogenic signatures, including Influenza A and other emerging betacoronaviruses, in which cytokine‐driven lung injury is a major determinant of morbidity and mortality. Given its favourable safety profile and the clear therapeutic window identified in this study, our data provide a robust scientific foundation for human clinical trials. Such trials should evaluate I3C not only as a COVID‐19 treatment but also as a strategic tool for pandemic preparedness, capable of stabilizing pulmonary integrity and preventing systemic hyperinflammation across a wide array of viral‐induced respiratory distress syndromes. Furthermore, our study establishes a viable framework for rapid preclinical evaluation of novel compounds against emerging respiratory viruses.

## Author Contributions

**Pocci Marco:** investigation, validation, visualization. **Biancolella Michela:** visualization, writing – review and editing. **Latini Andrea:** investigation, methodology, writing – review and editing. **Rizzacasa Barbara:** investigation, methodology, writing – review and editing. **Centofanti Federica:** investigation, methodology, writing – review and editing. **Mauriello Alessandro:** visualization, validation. **Servadei Francesca:** investigation, validation, methodology. **Scimeca Manuel:** investigation, methodology. **Claudia Filippone:** visualization, validation. **Bernadett Palyi:** investigation, methodology, validation. **Daniel Deri:** investigation, validation, methodology. **Pier Paolo Pandolfi:** conceptualization, funding acquisition, writing – review and editing. **Novelli Giuseppe:** conceptualization, visualization, writing – review and editing, funding acquisition, project administration. **Berenike Novak:** investigation, methodology, validation. **Zoltan Kis:** investigation, methodology, writing – review and editing.

## Funding

This work was supported by Italian Ministry of Research MURprogram PNRRM4‐C2‐I1.1 PRIN2022 “HECORES” (2022ZSLRPT) ; European Union – NextGenerationEU, PNRR M4‐C2‐1.4 (CN00000041); “UNDINE”, HORIZON‐HLTH‐2021‐DISEASE‐04‐07; Rome Foundation, Prot.317A/I.

## Conflicts of Interest

The authors declare no conflicts of interest.

## Supporting information


**Table S1:** List of primer sequences used for hamster inflammatory‐related genes.

## Data Availability

All data generated or analysed during this study are included in this published article. The study protocol and all data collected for the study, including raw data and analysis, will be made available upon request. Data will be made available after approval of a proposal and with a signed data access agreement.
