# Supplementary material for: Therapeutic Efficacy of Indole‐3‐Carbinol Against SARS‐CoV‐2‐Induced Acute Respiratory Distress Syndrome: A Dual Antiviral and Anti‐Inflammatory Approach in a Golden Syrian Hamster Model
Source: J Cell Mol Med. 2026 Jul 13;30(13):e71213. doi: 10.1111/jcmm.71213 (PMC13358549; doi:10.1111/jcmm.71213)
Supplement: Supplementary file 1 — Table S1: List of primer sequences used for hamster inflammatory‐related genes. [file JCMM-30-e71213-s001.docx]

**Supplementary Materials**

**Supplementary Table 1: List of primer sequences used for hamster inflammatory-related genes**

| GENE | FORWARD PRIMER (5'->3') | REVERSE PRIMER (5'->3') |
| --- | --- | --- |
| *TNF-α* | CCTCTCTCTAATCAGCCCTCTG | GAGGACCTGGGAGTAGATGAG |
| *IFNβ* | TTGTGCTTCTCCACTACAGC | GGCCATCCAGAGGAGCATAG |
| *CXCL10* | GCTATATTGGTCATCCAGCTGAGA | TGTCAACTGTGGATGAATGGC |
| *IL-6* | CCTGAAAGCACTTGAAGAATTCC | AGTGTGCTGTGCCTTAGCATACC |
| *ACTB* | ACTGCCGCATCCTCTTCCT | GTCATCACCATTGGCAACGA |
